# Epidemiology of Extended-Spectrum β-Lactamase Producing *Escherichia coli* in the Stools of Returning Japanese Travelers, and the Risk Factors for Colonization

**DOI:** 10.1371/journal.pone.0098000

**Published:** 2014-05-16

**Authors:** Kenichiro Yaita, Kotaro Aoki, Takumitsu Suzuki, Kazuhiko Nakaharai, Yukihiro Yoshimura, Sohei Harada, Yoshikazu Ishii, Natsuo Tachikawa

**Affiliations:** 1 Department of Infectious Diseases, Yokohama Municipal Citizen’s Hospital, Yokohama, Japan; 2 Department of Microbiology and Infectious Diseases, Toho University School of Medicine, Tokyo, Japan; 3 Department of Infectious Diseases, Cancer Institute Hospital, Japanese Foundation for Cancer Research, Tokyo, Japan; Institut National de la Recherche Agronomique, France

## Abstract

**Objective:**

Travel overseas has recently been considered a risk factor for colonization with drug-resistant bacteria. The purpose of this study was to establish the epidemiology and risk factors associated with the acquisition of drug-resistant bacteria by Japanese travelers.

**Methods:**

Between October 2011 and September 2012, we screened the stools of 68 Japanese returning travelers for extended-spectrum β-lactamase (ESBL) producing *Escherichia coli*. All specimens were sampled for clinical reasons. Based on the results, the participants were divided into an ESBL-producing *E. coli* positive group (18 cases; 26%) and an ESBL-producing *E. coli* negative group (50 cases; 74%), and a case-control study was performed. Microbiological analyses of ESBL-producing strains, including susceptibility tests, screening tests for metallo-β-lactamase, polymerase chain reaction amplification and sequencing of *bla*_CTX-M_ genes, multilocus sequence typing, and whole genome sequencing, were also conducted.

**Results:**

In a univariate comparison, travel to India was a risk factor (Odds Ratio 13.6, 95% Confidence Interval 3.0–75.0, p<0.0001). There were no statistical differences in the characteristics of the travel, such as backpacking, purpose of travel, interval between travel return and sampling stool, and duration of travel. Although 10 of 13 analyzed strains (77%) produced CTX-M-15, no ST131 clone was detected.

**Conclusion:**

We must be aware of the possibilities of acquiring ESBL-producing *E. coli* during travel in order to prevent the spread of these bacteria not only in Japan but globally.

## Introduction

In this era of globalization, drug-resistant bacteria are not only nosocomial, but may also be community-acquired. Acquiring extended-spectrum β-lactamase (ESBL)-producing bacteria during travel in developing countries is becoming a more serious threat. Moreover, colonization during overseas travel has become a risk factor for community-acquired infection (ex. urinary tract infection, bloodstream infection) due to ESBL-producing *Escherichia coli* [1] and the duration of colonization of ESBL-producing *E. coli* is longer than previously expected (10% of patients continued to carry at a 3-year follow-up) [2].

The reasons for the colonization of ESBL-producing bacteria during travel are expected to be exposure to a foreign environment (ex. poor-quality drinking water, and poor sewage disposal) [3] that is contaminated with drug-resistant organisms along with selection caused by antibiotic usage after the acquisition of such organisms [4], [5].

These situations cannot be overlooked even in developed countries such as Japan. Approximately 17 million Japanese now travel abroad each year, according to the Japan Tourism Agency (https://www.mlit.go.jp/kankocho/siryou/toukei/in_out.html). In particular, business trips between Japan and Asia (ex. India) have recently increased with the trend toward a global economy. However, no report has examined the relationship between Japanese travelers and ESBL-producing *E. coli* colonization, although infections and risk factors have been published. The purpose of our study was to clarify the epidemiology and risk factors associated with ESBL-producing *E. coli* colonization in Japanese returning travelers.

## Patients and Methods

This was a single-center research conducted by the Department of Infectious Diseases of Yokohama Municipal Citizen’s Hospital, a 650-bed tertiary care medical center. Between October 2011 and September 2012 (one year), returning travelers with Japanese citizenship whose stool specimens were sampled for clinical reasons (ex. traveler’s diarrhea, fever after travel) were included in this study. Infectious disease physicians selected these participants from patients presenting with health problems that may have been related to recent travels. Retrospectively, we checked the electronic medical charts of participants and classified their factors as follows: age, sex, the symptoms (diarrhea, fever, abdominal pain), ESBL-producing *E. coli* colonization, travel destination (India, Asia except India, Oceania, Africa, North America, Central America, South America, Europe), backpacking travelers, purpose of travel (vacation, business/education/volunteer work, visiting friends and relatives (VFR)), the interval between travel return and stool sampling (>10 days or not), duration of travel (>10 days or not), antibiotic treatment before the visit to our clinic. Diarrhea was defined as the passage of 3 or more unformed stools in a day. Fever was defined as 37.5°C, or more, of axillary body temperature at least once before consultation to our clinic.

We selectively cultured stool samples collected from participants for ESBL producing Enterobactriacae using ChromID ESBL ager (SYSMEX bioMérieux Co., Ltd., Tokyo, Japan) as the initial screen test. We selected colonies that were consistent with producing ESBL for further identification. For the identification of *E. coli*, we referred to the combination of the biochemical results [6]. Susceptibility was assessed by a broth microdilution method using Dry Plate (Eiken Cheminal Co., Ltd., Tokyo, Japan) according to the guidelines of the Clinical Laboratory Standards Institute [7]. ESBL production was confirmed by using the combination disk method according to the Clinical and Laboratory Standards Institute criteria [8]. In addition, the dipicolinic acid-based (DPA) disk method was used for evaluation of the production of metallo-β-lactamase [9].

*E. coli* harboring ESBL were subjected to PCR amplification and sequencing of *bla*_CTX-M_ genes and multilocus sequence typing (MLST). *bla*_CTX-M_ genes were screened as described previously [10]. For PCR amplification of *bla*_CTX-M_, we used three sets of PCR primers: IS*Ecp1*U1-CTX-M-2-group, IS*Ecp1*U1-CTX-M-3-group, and IS*Ecp1*U1-CTX-M-9-group. The forward primer is common in three sets: IS*Ecp1*U1 (5′-AAA AAT GAT TGA AAG GTG GT-3′) [11]. The reverse primers were as follows: 5′-TTA TGG CCT GGT ATG CGC AAG-3′ for the CTX-M-2 group, 5′-TTA TGG CCT GGT ATG CGC AAG-3′ for the CTX-M-3 group, and 5′-TGA GGG CTT TAT TGT AGG TG-3′ for the CTX-M-9 group. MLST was performed for seven housekeeping genes (*adk*, *fumC*, *gyrB*, *icd*, *mdh*, *purA*, and *recA*), and we collated them with MLST databases at the ERI, University College Cork web site (http://mlst.ucc.ie/mlst/dbs/Ecoli).

In addition, whole genome sequencing (WGS) was performed to screen for the presence of additional antimicrobial resistance genes other than *bla*_CTX-Ms_. The sequencing library was prepared with the Nextera XT DNA Sample Prep Kit (Illumina, San Diego, CA, USA) and sequencing was performed using a MiSeq sequencer (Illumina) in a 2×300-bp paired-end run. Genome assembly was performed using the A5-miseq assembly pipeline [12]. Antimicrobial resistance genes were identified from WGS data using the ResFinder 2.1 Website (http://cge.cbs.dtu.dk/services/ResFinder/).

We divided patients into an ESBL-producing *E. coli* positive group (returning travelers in whose stools ESBL-producing *E. coli* were detected) and an ESBL-producing *E. coli* negative group (returning travelers in whose stools ESBL-producing *E. coli* were not detected) and performed a case-control study between these two groups. All statistical analyses were performed with EZR (Saitama Medical Center, Jichi Medical University), which is a graphical user interface for R (The R Foundation for Statistical Computing, version 2.14.1) [13]. Categorical data were tested using a Fisher’s exact test, and continuous data were tested using a student’s t test. A *p*-value <0.05 was considered to be statistically significant.

The Yokohama Municipal Citizen’s Hospital Research Ethics Committee approved the study. The ethics committee waived the need for the written informed consent for using participant’s samples and analyzing clinical case records because of preserved anonymity. However, from the participants in the ESBL-producing *E. coli* positive group, the written informed consent was obtained for performing the advanced microbiological analysis and reporting of the detailed information, in accordance with the committee’s counsel.

## Results

Of 179 returning Japanese travelers who visited our clinic between October 2011 and September 2012, 68 patients whose stools were cultured, participated in this study. The mean age was 34.5 (range, 0 to 76) years, and 48 patients (71%) were male. The mean interval between the day of return to Japan and the day their stools were sampled was 5.5 (range, 0 to 67) days. The mean duration of travel was 16.7 (range, 2 to 248) days. Of the total, 57 patients (84%) had traveled to Asian countries and 14 patients (21%) to India which was the most frequent travel destination. Eighteen (26%) of the participants were found to be positive for ESBL-producing *E. coli* (ESBL-producing *E. coli* positive group), and 50 (74%) were found to be negative for ESBL-producing *E. coli* in their stools (ESBL-producing *E. coli* negative group).

The univariate comparisons of variables between the ESBL-producing *E. coli* positive group and the ESBL-producing *E. coli* negative group are summarized in Table 1. Of the 18 travelers who traveled for less than 10 days, 14 had ESBL-producing *E. coli* in their stools. Only 4 travelers in the ESBL-producing *E. coli* positive group had been prescribed antibiotics before consultation to our clinic.

**Table 1:** The characteristics of ESBL-producing *E. coli* positive/negative groups.

	ESBL-producing *E. coli*positive group (n = 18)	(%)	ESBL-producing *E. coli*negative group (n = 50)	(%)	*p* value
Age, mean ± SD (year)	33.9±12.9	−	34.7±15.6	−	0.851)
Sex (male)	11	(61)	37	(74)	0.37^2)^
Symptoms					
Diarrhea	18	(100)	43	(86)	0.18^2)^
Fever (> = 37.5°C)	8	(44)	32	(64)	0.17^2)^
Abdominal pain	11	(61)	34	(68)	0.77^2)^
Travel destination					
India	10	(56)	4	(8)	<0.0001^2)^
Asia except India	9	(50)	36	(72)	0.15^2)^
Oceania	0	(0)	1	(2)	1.00^2)^
Africa	0	(0)	5	(10)	0.32^2)^
North America	0	(0)	2	(4)	1.00^2)^
Central America	0	(0)	1	(2)	1.00^2)^
South America	0	(0)	1	(2)	1.00^2)^
Europe	1	(6)	2	(4)	1.00^2)^
Backpacking travelers	1	(6)	3	(6)	1.00^2)^
Purpose of travel					
Vacation	10	(56)	29	(58)	1.00^2)^
Business/Education/ Volunteer work	7	(39)	20	(40)	1.00^2)^
VFR	1	(6)	4	(8)	1.00^2)^
Interval between travel return andsampling stool >10 days	2	(11)	7	(14)	1.00^2)^
Duration of travel >10 days	4	(22)	13	(26)	1.00^2)^
Antibiotics treatment beforeconsultation to our clinic	4	(22)	12	(24)	1.00^2)^

1) Student’s t-test, ^2)^ Fisher’s exact test.

ESBL = extended-spectrum β-lactamase; SD = standard deviation; VFR = visiting friends and relatives;

India as a travel destination was a significant risk factor for the occurrence of ESBL-producing *E. coli* in the stools (Odds Ratio 13.6, 95% Confidence Interval (CI) 3.0–75.0, p<0.0001). Of 14 travelers to India, 10 travelers carried ESBL-producing *E. coli*. Among these travelers to India, there were no statistical differences in other travel factors such as backpacking travelers, purpose of travel, interval between travel return and sampling stool, duration of travel and antibiotics treatment before consultation to our clinic (Table 2).

**Table 2:** The characteristics of ESBL-producing *E. coli* positive/negative groups in travelers to India.

	ESBL-producing *E. coli*positive group (n = 10)	(%)	ESBL-producing *E. coli*negative group (n = 4)	(%)	*p* value
Age, mean ± SD (year)	31.7±8.8	–	35.0±17.0	–	0.641)
Sex (male)	6	(60)	4	(100)	0.25^2)^
Symptoms					
Diarrhea	10	(100)	3	(75)	0.29^2)^
Fever (> = 37.5°C)	4	(40)	4	(100)	0.08^2)^
Abdominal pain	6	(60)	2	(50)	1.00^2)^
Backpacking travelers	1	(10)	1	(25)	0.51^2)^
Purpose of travel					
Vacation	6	(60)	3	(75)	1.00^2)^
Business/Education/ Volunteer work	4	(40)	1	(25)	1.00^2)^
VFR	0	(0)	0	(0)	−
Interval between travel returnand sampling stool >10 days	1	(10)	0	(0)	1.00^2)^
Duration of travel >10 days	3	(30)	3	(75)	0.25^2)^
Antibiotics treatment beforeconsultation to our clinic	3	(30)	1	(25)	1.00^2)^

1) Student’s t-test, ^2)^ Fisher’s exact test.

ESBL = extended-spectrum β-lactamase; SD = standard deviation; VFR = visiting friends and relatives;

Table S1 lists the details of ESBL-producing *E. coli* positive travelers including microbiological findings. Two participants declined to be listed in Table S1. No strain belonged to CC131 (including ST131). Of the 13 analyzed strains, 10 carried *bla*_CTX-M-15_ and 8 carried *bla*_TEM-1_.

In Table S2, we summarized the minimum inhibitory concentrations of the strains of ESBL-producing *E. coli.* No strain was resistant to carbapenems, and 5 strains were resistant to quinolones. The DPA disk method and WGS also revealed no strain producing carbapenemases including metallo-β-lactamase.

## Discussion

In this study, the prevalence of the colonization of ESBL-producing *E. coli* in returning Japanese travelers was 26%, and this value was compatible with past reports (23–30%) [14]–[17]. We did not compare travelers with non-travelers as a control group. However, a previous study showed that ESBL-producing *E. coli* is colonized in only 5% of the stools of healthy adults in Japan [18]. This result is similar to a previous study in Canada (4% of non-travelers had ESBL-producing *E. coli*) [15]. From these results, traveling abroad is considered a risk factor for the acquisition of ESBL-producing *E. coli*.

In particular, traveling to India was a significant risk factor for ESBL-producing *E. coli* colonization. This result is compatible with the following published reports [1], [14], [15], [19], [20]. Tängdén et al. reported that 88% of travelers to India acquired ESBL-producing *E. coli,* and the symptoms of gastroenteritis were considered a risk of the acquisition of ESBL-producing *E. coli* [14]. Laupland et al. revealed that traveling to India was an important risk factor for community-acquired ESBL-producing *E. coli* infection (Relative Risk 145.6) [1]. This is because India has a higher rate of ESBL-producing *E. coli* contamination. Hawser et al. established that the frequency of ESBL-producing strains of *E. coli* from patients with intra-abdominal infections in India were the highest (79%) of all Asian-Pacific regions (28%) [21]. In our study, diarrhea-suffering travelers in the ESBL-producing *E. coli* positive group (100%) numbered more than those in the ESBL-producing *E. coli* negative group (86%), although there was no statistical significance between the groups. From these results, we concluded that those in the ESBL-producing *E. coli* positive group were more likely to be exposed to food or water contaminated with gastrointestinal pathogenic bacteria (the cause of traveler’s diarrhea) and ESBL-producing *E. coli*. A high prevalence of ESBL-producing *E. coli* in communities of India and a lack of proper hygienic handling of food and/or water [3], are considered to be the factors promoting ESBL-producing *E. coli* colonization in travelers to India [14].

Travel to other regions of Asia (except India) and Africa is also considered to be a risk factor for the acquisition of ESBL-producing *E. coli* [1]. However, there were no other statistically significance in our study. The reason for no ESBL-producing *E. coli* positive cases in returning travelers from Africa might have been the small size of our samples. Based on the results of past reports [1], [14], [15], [17] combined with the results of our study, travel to Asia (except India) and Africa is not considered a high risk compared with India. The data concerning the ratio of ESBL-producing strains in *E. coli* in Asia and Africa were limited, but were reported as follows (these data are calculated from specimens, isolated from intra-abdominal infections); China (55%), Thailand (50.8%), Vietnam (34.4%), and South Africa (7.6%) [21], [22]. No country has reported a higher prevalence of ESBL-producing *E. coli* than India. This may explain the higher risk of colonization in India.

There were no risk factors connected with the characteristics of travel (backpacking, purpose of travel, interval between travel return and sampling stool, duration of travel, and antibiotic treatment before consultation to our clinic) in this study, which agreed with a previous report [14]. The acquisition of ESBL-producing *E. coli* could happen even within a short stay such as a business trip (in particular, to India). And in Table S1, there were only 4 travelers who had in-travel consultation and/or admission to foreign hospitals. As the trend toward the globalization of business increases, the number of business trips to Asian countries (especially India) is expected to increase over the next few decades. Such business trips can be opportunities to transport drug-resistant bacteria from developing countries to other countries. This result should interest clinical physicians the most. We always inquire as to the “travel history” of our patients in order to detect tropical diseases. However, in addition, we should consider colonization/infection with drug-resistant bacteria [1], [23]. Contact precaution should be a routine recommendation for returning travelers from the viewpoint of infection control.

In our study, most of the strains produced CTX-M-15 (included in the CTX-M-1 group), however, sequence types were diverse. In India, it is well known that CTX-M-15-producing *E. coli* is predominant [4], [24]. On the other hand, the CTX-M-9-group is now spreading in Japan followed by the CTX-M-2 and the CTX-M-1-groups [25]–[27]. Some studies have already shown that most of the strains of *E. coli* that the patients acquired in lndia had the CTX-M-15 enzyme [1], [17], [20]. Interestingly, there is a diversity of sequence types and no strain included in CC131 (ST131 belongs) was detected in this study. Although the ST131 clone is spreading globally and is dominant [28], [29], other studies [15], [16], [30] have reported that other strains are also producing CTX-M-15. In Pitout’s report, half of the strains producing CTX-M-15 of travel-related ESBL-Producing *E. coli* Isolates did not belong to ST131 [30].

Our study had some limitations. First, since this was a retrospective case-control study, we could not distinguish between colonization before travel and its acquisition during travel. Second, we could not check the stools of all returning travelers who visited our clinic. The reason for sampling the participant’s stools for this study was clinically necessity (ex. detection of the organisms causing traveler’s diarrhea, a part of the fever-workup). There is the possibility that the results would change, if the stools of all returning travelers were collected. This inclusion criteria might have negated the rate of diarrhea-suffering travelers between ESBL-producing *E. coli* positive groups and negative groups, which would have been different from the past study [14].

In conclusion, travel in India is a risk factor for ESBL-producing *E. coli* colonization in Japanese travelers, even for short durations of travel. In addition, the characteristics of travel might not be related to the risk of colonization. The results of this study have alerted us to the necessity of considering ESBL-producing *E. coli* beyond situations of only hospital-acquired/community-acquired to include situations of “travel-acquired.” The spread of these strains should be prevented globally, and not only in Japan.

## Supporting Information

Table S1 **The list of ESBL-producing** ***E. coli*** **colonizing travelers, characteristics of the travel, sequence types of MLST and ESBL encoding genes.**
(XLS)

Table S2 **Minimum inhibitory concentrations of the strains of ESBL-producing** ***E. coli*** **.**
(XLS)
